# Adiponectin Isoforms Differentially Affect Gene Expression and the Lipidome of Primary Human Hepatocytes

**DOI:** 10.3390/metabo4020394

**Published:** 2014-05-23

**Authors:** Josef Wanninger, Gerhard Liebisch, Kristina Eisinger, Markus Neumeier, Charalampos Aslanidis, Lisa Voggenreiter, Rebekka Pohl, Thomas S. Weiss, Sabrina Krautbauer, Christa Buechler

**Affiliations:** 1 Department of Internal Medicine I, Regensburg University Hospital, Regensburg 93042, Germany; E-Mails: josef.wanninger@gmx.net (J.W.); kristina.eisinger@klinik.uni-regensburg.de (K.E.); neumeier.markus@web.de (M.N.); lisa.voggenreiter@klinik.uni-regensburg.de (L.V.); rebekka.pohl@klinik.uni-regensburg.de (R.P.); sabrina.krautbauer@klinik.uni-regensburg.de (S.K.); 2 Institute for Clinical Chemistry and Laboratory Medicine, Regensburg University Hospital, Regensburg 93042, Germany; E-Mails: gerhard.liebisch@klinik.uni-regensburg.de (G.L.); charalampos.aslanidis@klinik.uni-regensburg.de (C.A.); 3 University Children Hospital (KUNO), Regensburg University Hospital, Regensburg 93042, Germany; E-Mail: thomas.weiss@klinik.uni-regensburg.de

**Keywords:** ceramide, phospholipids, gene expression, trimer, lipidomic

## Abstract

Adiponectin (APN) exerts multiple beneficial effects in obesity and protects from liver injury. Different APN isoforms circulate in serum, and here, the effect of low molecular weight (LMW) and higher molecular weight (HMW) APN on primary human hepatocytes (PHH) has been analyzed. APN is not detected in hepatocyte lysates; levels are strongly increased by HMW-APN, but not by LMW-APN, suggesting the distinct uptake/degradation of APN isoforms by PHH. Several genes with a role in fibrosis, glucose and lipid metabolism known to be regulated by HMW-APN are not affected by the LMW-isoform. Follistatin is reduced by HMW-APN and induced by LMW-APN in supernatants of PHH. Fibroblast growth factor 21 is repressed by both isoforms. Cellular triglycerides and cholesterol levels are not reduced by APN. Total phospholipids, including plasmalogens and sphingomyelins, are not changed upon APN incubation, while distinct species are either induced or repressed. Unexpectedly, total ceramide is increased by LMW-APN. Current data show that APN isoforms differentially affect hepatocyte gene expression, but do not grossly alter the hepatocyte lipidome.

## 1. Introduction

Obesity is a major risk factor for metabolic diseases, including non-alcoholic fatty liver disease (NAFLD) [1]. Higher fat mass is associated with reduced circulating adiponectin (APN), which, by itself, exerts multiple beneficial activities, thus counteracting adverse metabolic effects. This adipokine exerts insulin-sensitizing, anti-steatotic and anti-fibrotic activities [2]. Various hepatic genes involved in mitochondrial function, fibrosis, lipid and glucose metabolism are regulated by this adipokine [3,4,5,6,7,8,9]. APN also protects hepatocytes from apoptosis by antagonizing free fatty acid-induced CD95 expression [10]. This adipokine further antagonizes increased bile acid synthesis in NAFLD, which contributes to hepatocyte death [11]. In NAFLD patients, systemic APN is decreased and reversely correlates with the non-alcoholic steatohepatitis activity score, CD95 mRNA levels, hepatocellular apoptosis and serum bile acids [11].

Ceramide is increased in the steatotic liver of obese ob/ob and high-fat diet-fed mice. APN lowers ceramide and dihydroceramide species and, thereby, ameliorates hepatic insulin sensitivity. Ceramide is converted to sphingosine by adiponectin receptor-mediated ceramidase activity. Elevated ceramide in palmitate-incubated rat hepatoma cells is also normalized by APN, suggesting a direct effect on liver parenchymal cells [12]. 

APN has, however, no effect on ceramide levels in lean animals [12]. In the liver of APN-deficient mice, ceramide is not induced, and hexosyl ceramide is even reduced [13]. This suggests that adiponectin receptor-associated ceramidase is specifically activated in fatty liver.

APN activity depends on its oligomeric form, which ranges in size from a 90-kDa trimer (low molecular weight, LMW) to 180-kDa hexamers (medium molecular weight, MMW) and larger higher molecular weight (HMW) forms [14,15]. These oligomeric complexes circulate in blood and have partly diverging biological activities. Hexameric and higher order oligomers, but not trimeric forms, stimulate nuclear factor kappa B [15]. Trimeric, but not higher order, multimers have anti-inflammatory effects in monocytes [16]. In subcutaneous and visceral adipose tissues, APN isoform-specific anti-lipolytic effects are actually modified by obesity [17]. Trimeric APN is supposed to more effectively lower hepatocyte glucose production [18]. In serum, HMW-APN is modestly more abundant than LMW-APN, while MMW-APN is about 20% to 50% lower [19,20].

Most studies suggest that HMW-APN represents the physiologically most important form, and only this isoform correlates with visceral fat mass, liver steatosis and insulin sensitivity [21,22,23]. Hypothesizing that LMW-APN affects primary human hepatocyte function, the effect of this APN isoform on the expression of several genes and the hepatocyte lipidome has been investigated herein. 

Here, recombinant human LMW-APN produced in insect cells and recombinant human HMW-APN expressed in a mouse cell line [16] have been used to study the effects of these APN isoforms on the expression of genes already described to be regulated by HMW-APN in primary human hepatocytes (PHH) [5,7,8]. Fibroblast growth factor 21 (FGF21) turned out to exert its metabolic function by the induction of adiponectin [24], and therefore, the regulation of hepatocyte FGF21 by APN isoforms has been determined. Furthermore, various lipid classes have been measured to identify species affected by these APN isoforms. 

## 2. Results and Discussion

### 2.1. APN Isoforms in Hepatocyte Lysates

In monocytes, 10 µg/mL of HMW-APN and 1 and 2 µg/mL of LMW-APN have been shown to be biologically active [16]. Therefore, 10 µg/mL of HMW-APN and 2 µg/mL of LMW-APN have been used in the current experiments. Primary human hepatocytes could not be obtained in large quantities, and therefore, only one concentration of each isoform has been used.

**Figure 1:**
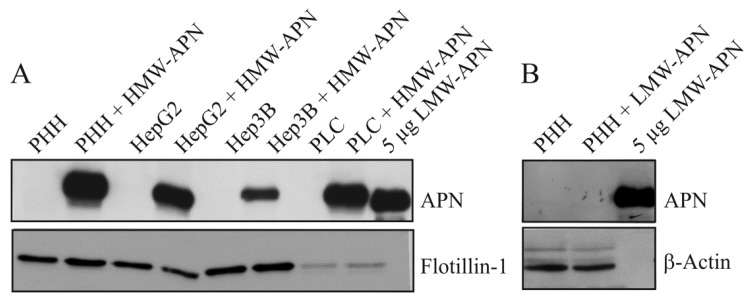
Adiponectin (APN) in hepatocyte lysates. (**A**) Primary human hepatocytes (PHH), HepG2, Hep3B and PLC/PRF/5 hepatomacell lines were cultivated in the presence of 10 µg/mL of high molecular weight (HMW)-APN for 24 h. APN and Flotillin-1 were analyzed by immunoblot, and APN was detected in lysates of HMW-APN-incubated PHH. Recombinant low molecular weight (LMW)-APN is shown as the positive control; (**B**) PHH were cultivated in the presence of 2 µg/mL of LMW-APN for 24 h. APN and β-actin were analyzed by immunoblot. APN is not detected in cell lysates. Recombinant LMW-APN is shown as the positive control.

Hepatocytes were cultivated for 24 h in medium with or without the addition of 10 µg/mL of HMW-APN or 2 µg/mL of LMW-APN. Immunoblot analysis shows that APN is not detectable in the lysates of non-treated control cells and LMW-APN-incubated PHH. A representative result of three independent experiments is shown in Figure 1A,B. APN is found in lysates of hepatocyte cell lines and PHH where HMW-APN has been added (Figure 1A). APN was measured by ELISA in lysates of PHH incubated with increasing concentrations of APN isoforms. Whereas lysate APN increases with higher concentrations of HMW-APN, as has already been shown [25], it is not detected in PHH incubated with 5, 10 or 20 µg/mL of LMW-APN (data not shown). This may reflect differences in the uptake and/or degradation of the two isoforms studied. In mice, most of the serum adiponectin is cleared by the liver, and trimeric APN is removed much faster than HMW-APN [26]. Although there are data indicating that clearance is linked to bioactivity, there is no final proof of this assumption [26].

### 2.2. Effect of APN Isoforms on Genes/Proteins Involved in Fibrosis

Our group has shown that HMW-APN upregulates the pro-fibrotic and anti-steatotic protein, activin A, and suppresses its natural inhibitor, follistatin [7,27]. HMW-APN also increases MMP-9 and TIMP-1, which participate in extracellular matrix degradation [8,28]. Simultaneous upregulation of MMPs and its inhibitors by adiponectin has also been reported in dermal fibroblasts [29]. Further, cAMP has been shown to induce MMP-9 and TIMP-1 in rat hepatocytes [30]. This suggests that MMP-9 activity is tightly regulated. Of note, MMP-9 activity is increased by HMW-APN, demonstrating that induction of TIMP-1 does not fully block enzyme function [8]. 

**Figure 2:**
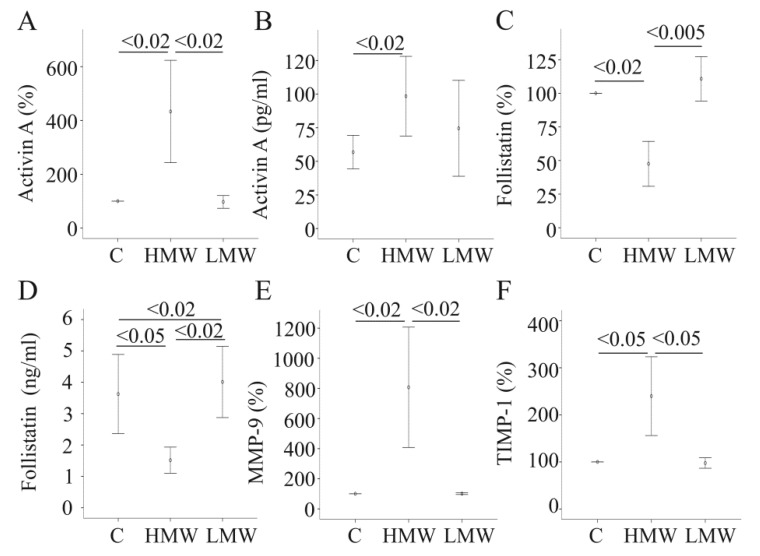
The effect of adiponectin (APN) isoforms on genes/proteins involved in fibrosis. (**A**) Activin A mRNA expression was determined in primary human hepatocytes (PHH) of four different donors, either cultivated in the presence of 10 µg/mL of HMW-APN or 2 µg/mL of LMW-APN for 24 h. (**B**) Activin A protein was measured by ELISA in the supernatants of cells described in (A); data of five experiments are shown. (**C**) Follistatin mRNA expression in the cells described in (A), data of four experiments are shown; (**D**) Follistatin protein was measured by ELISA in the supernatants of cells described in (A); data of three experiments are shown. (**E**) The expression of MMP-9 mRNA in the cells described in (A); data of four experiments are shown. (**F**) The expression of TIMP-1 mRNA in the cells described above; data of four experiments are shown. Data on mRNA expression are given as % of control cultivated cells. Numbers within the graphs indicate *p*-values.

Here, it has been tested whether LMW-APN also affects the mRNA levels of these genes. However, none of these genes is regulated by LMW-APN (Figure 2A,C,E,F). Activin A and follistatin were measured in the cell supernatants by ELISA. In accordance with the mRNA data, HMW-APN increases activin A and decreases follistatin, as has already been shown [7] (Figure 2B,D). LMW-APN, though, has no effect on activin A protein increases follistatin protein in the supernatants (Figure 2 B,D). These findings suggest that LMW-APN may even antagonize HMW-APN-mediated induction of activin A activity by increasing the level of its inhibitor, follistatin.

### 2.3. Effect of APN Isoforms on Genes Involved in Glucose and Lipid Metabolism

**Figure 3:**
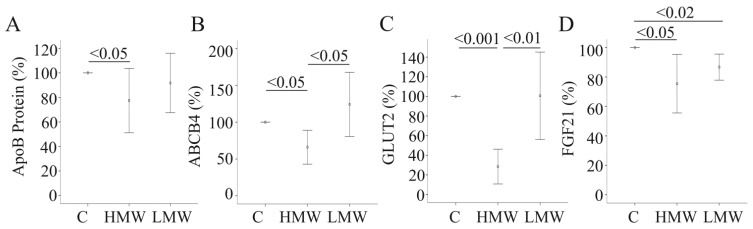
Effect of adiponectin (APN) isoforms on genes/proteins involved in glucose and lipid metabolism. (**A**) Apolipoprotein B-100 was determined by ELISA in the supernatants of primary human hepatocytes (PHH) of six different donors, either cultivated in the presence of 10 µg/mL of HMW-APN or 2 µg/mL of LMW-APN for 24 h. (**B**) The expression of ABCB4 was determined in PHH of five different donors, either cultivated in the presence of 10 µg/mL of HMW-APN or 2 µg/mL of LMW-APN for 24 h. (**C**) The expression of GLUT2 was determined in PHH of six different donors, either cultivated in the presence of 10 µg/mL of HMW-APN or 2 µg/mL of LMW-APN for 24 h; (**D**) The expression of FGF21 was determined in PHH of five different donors, either cultivated in the presence of 10 µg/mL of HMW-APN or 2 µg/mL of LMW-APN for 24 h. Data are shown as % of control cultivated cells. Numbers within the graphs indicate *p*-values.

HMW-APN reduces the mRNA expression and protein levels of apolipoprotein B-100, the major apolipoprotein in very low density lipoprotein, mRNA expression of the phosphatidylcholine transporter, ABCB4, and the facilitative glucose transporter, GLUT2 [5]. Again, LMW-APN does not affect mRNA levels of these proteins, and apolipoprotein B-100 protein is not altered in the supernatants (Figure 3A–C and data not shown). These findings do not indicate that LMW-APN is biologically inactive in PHH, but suggest that this isoform exerts effects distinct from HMW-APN.

### 2.4. Effect of APN Isoforms on Fibroblast Growth Factor 21

FGF21 is produced in several organs, including the liver, and is a potent anti-diabetic protein [24]. FGF21 induces APN, which mediates part of its beneficial effects [24]. Here, it was analyzed whether APN itself regulates hepatocyte FGF21. FGF21 is reduced by both APN isoforms (Figure 3D). Systemic FGF21 is not altered in APN knock-out mice, excluding APN as a principal regulatory factor [24,31]. Anyway, LMW-APN and HMW-APN both lower FGF21 mRNA in PHH. This does not contradict the findings in animal studies [24,31], because lower production in liver parenchymal cells does not mandatorily cause reduced circulating FGF21. Further, changes in mRNA expression may not necessarily result in reduced protein production, and FGF21 protein levels have not been determined herein.

### 2.5. Effect of APN Isoforms on Triglycerides and Cholesterol

**Figure 4:**
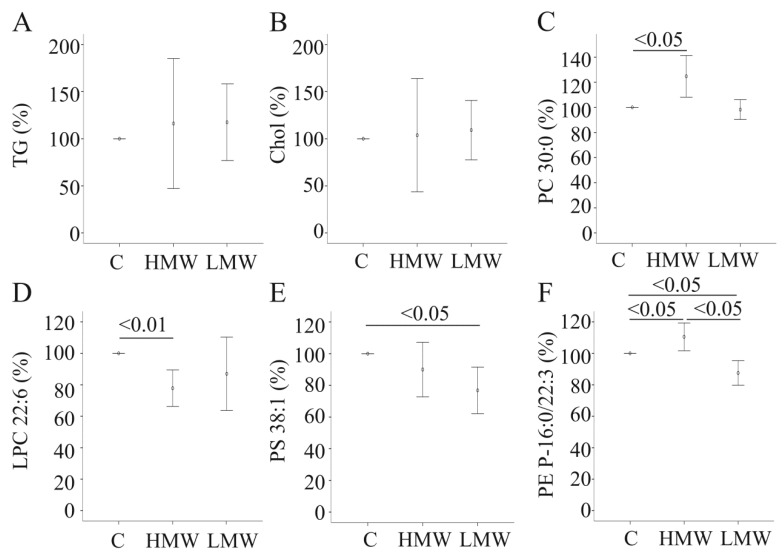
Effect of adiponectin (APN) isoforms on cellular lipids. (**A**) Triglycerides (TG) were determined in cell lysates of PHH of five different donors, either cultivated in the presence of 10 µg/mL of HMW-APN or 2 µg/mL of LMW-APN for 24 h. (**B**) Cholesterol (Chol) in cell lysates described in (A). (**C**) Phosphatidylcholine (PC) 30:0 in cell lysates of PHH of four different donors, either cultivated in the presence of 10 µg/mL of HMW-APN or 2 µg/mL of LMW-APN for 24 h. (**D**) Lysophosphatidylcholine (LPC) 22:6 in cells described in (C); (**E**) Phosphatidylserine (PS) 38:1 in cell lysates described in (C). (**F**) Phosphatidylethanolamine-based plasmalogens (PE P)-16:0/22:3 in cell lysates described in (C). Values are given as % of control cultivated cells. Numbers within the graphs indicate p-values.

APN knock-out mice fed a standard chow do not develop liver steatosis, and total cholesteryl ester concentration is even modestly reduced in the liver [13]. In accordance, APN isoforms do not reduce triglyceride levels in PHH (Figure 4A). Total cholesterol is also not changed in PHH of five different donors incubated with APN isoforms for 24 h (Figure 4B), and lower cholesteryl ester in the mice [13] is most likely not a direct effect of APN deficiency.

### 2.6. Effect of APN Isoforms on Phosphatidylcholine and Lysophosphatidylcholine

APN isoforms do not alter total, monounsaturated (MUFA), polyunsaturated (PUFA) and saturated phosphatidylcholine (PC) species in PHH of four different donors within 24 h of cultivation (data not shown). PC 30:0 (0.23 ± 0.01 nmol/mg in control-, 0.28 ± 0.03 nmol/mg in HMW-APN- and 0.23 ± 0.03 nmol/mg in LMW-APN-incubated cells) is raised by HMW-APN (Figure 4C) and PC 32:2 (0.15 ± 0.05 nmol/mg in control-, 0.16 ± 0.06 nmol/mg in HMW-APN- and 0.17 ± 0.04 nmol/mg in LMW-APN-incubated cells) and PC 38:6 (7.42 ± 3.52 in control-, 6.87 ± 4.24 in HMW-APN- and 8.02 ± 3.98 in LMW-APN-incubated cells) by LMW-APN. Lysophosphatidylcholine (LPC) species 22:6 (0.12 ± 0.07 nmol/mg in control-, 0.10 ± 0.07 nmol/mg in HMW-APN- and 0.12 ± 0.10 nmol/mg in LMW-APN-incubated cells) is reduced by HMW-APN (Figure 4D). These data show that APN isoforms alter the level of individual PC and LPC species. The biological role of these individual lipids has not been evaluated to our knowledge, so far, thus hindering speculations about their physiological function.

### 2.7. Effect of APN Isoforms on Phosphatidylethanolamine and Phosphatidylserine

MUFA phosphatidylethanolamine (PE) (31.94 ± 18.86 nmol/mg in control-, 28.00 ± 25.26 nmol/mg in HMW-APN- and 35.44 ± 21.10 nmol/mg in LMW-APN-incubated cells) is higher in LMW-APN-incubated PHH, and PE 32:1 (0.06 ± 0.05 nmol/mg in control-, 0.07 ± 0.06 nmol/mg in HMW-APN- and 0.08 ± 0.05 nmol/mg in LMW-APN-incubated cells), PE 36:1 (1.06 ± 0.66 nmol/mg in control-, 0.88 ± 0.45 nmol/mg in HMW-APN- and 1.12 ± 0.64 nmol/mg in LMW-APN-incubated cells) and PE 42:7 (0.06 ± 0.02 nmol/mg in control-, 0.06 ± 0.02 nmol/mg in HMW-APN- and 0.08 ± 0.01 nmol/mg in LMW-APN-incubated cells) are significantly increased. HMW-APN does not affect PE species levels (data not shown). MUFA PE species are even elevated in the liver of APN knockout mice [13], suggesting that metabolites, besides APN, have a more prominent physiological role in the regulation of hepatic MUFA PE levels. Regarding phosphatidylserine (PS) species, PS 38:1 (0.03 ± 0.01 nmol/mg in control-, 0.03 ± 0.01 nmol/mg in HMW-APN- and 0.02 ± 0.01 nmol/mg in LMW-APN-incubated cells) is reduced by LMW-APN (Figure 4E).

### 2.8. Effect of APN Isoforms on Plasmalogens

Phosphatidylethanolamine-based plasmalogens (PE P) with 16:0, 18:0 and 18:1 vinyl ether bonds have been measured. PE P-16:0/20:3 (0.012 ± 0.003 nmol/mg in control-, 0.015 ± 0.005 nmol/mg in HMW-APN- and 0.015 ± 0.004 in LMW-APN-incubated cells) is raised by HMW-APN and PE P-18:1/22:4 (0.010 ± 0.007 nmol/mg in control-, 0.013 ± 0.003 nmol/mg in HMW-APN- and 0.014 ± 0.002 nmol/mg in LMW-APN-incubated cells) by LMW-APN. PE P‑16:0/22:3 (0.021 ± 0.002 nmol/mg in control-, 0.023 ± 0.004 nmol/mg in HMW-APN- and 0.018 ± 0.002 nmol/mg in LMW-APN-incubated cells) is lower in LMW-APN-incubated PHH and increased by the HMW isoform (Figure 4F). PE P-18:0/22:4 (0.020 ± 0.006 nmol/mg in control-, 0.023 ± 0.007 nmol/mg in HMW-APN- and 0.023 ± 0.007 nmol/mg in LMW-APN-incubated cells) and 18:1/20:4 (0.038 ± 0.021 nmol/mg in control-, 0.045 ± 0.033 nmol/mg in HMW-APN- and 0.048 ± 0.025 nmol/mg in LMW-APN-incubated cells) are induced by the LMW form. PE P-18:1/18:3 (0.011 ± 0.002 nmol/mg in control-, 0.012 ± 0.002 nmol/mg in HMW-APN- and 0.012 ± 0.001 nmol/mg in LMW-APN-incubated cells) is raised by both isoforms.

Total PE P levels are not changed (data not shown). Plasmalogens protect from oxidative stress [32] and are mostly increased by APN. Whether this contributes to the beneficial effects of APN needs to be addressed by additional studies.

### 2.9. Effect of APN Isoforms on Sphingomyelin and Ceramides

**Figure 5:**
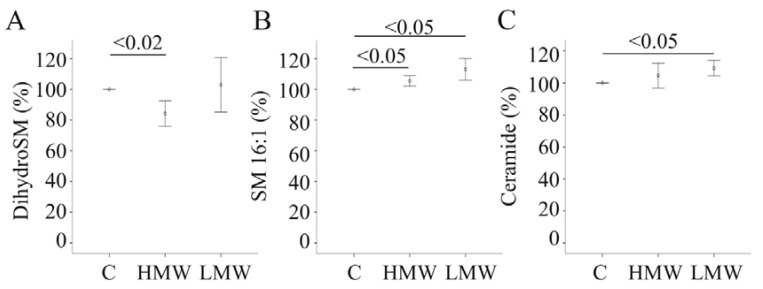
The effect of adiponectin (APN) isoforms on cellular sphingomyelin (SM) and ceramide (Cer). (**A**) Dihydrosphingomyelin (dihydroSM) in cell lysates of PHH of four different donors, either cultivated in the presence of 10 µg/mL of HMW-APN or 2 µg/mL of LMW-APN for 24 h. (**B**) SM16:1 in these cell lysates. (**C**) Cer in these cell lysates. Values are given as % of control cultivated cells. Numbers within the graphs indicate *p*-values.

Total saturated, total MUFA and PUFA sphingomyelin (SM) species are neither affected by LMW-APN nor HMW-APN. Dihydrosphingomyelin (0.37 ± 0.22 nmol/mg in control-, 0.30 ± 0.13 nmol/mg in HMW-APN- and 0.38 ± 0.22 nmol/mg in LMW-APN-incubated cells) is reduced in PHH incubated with HMW-APN (Figure 5A). Regarding individual SM species, SM 16:1 (0.12 ± 0.05 nmol/mg in control-, 0.13 ± 0.05 nmol/mg in HMW-APN- and 0.13 ± 0.05 nmol/mg in LMW-APN-incubated cells) is increased by both isoforms (Figure 5B). Up to now, neither the pathways involved herein nor specific functions of these lipids have been described.

HMW-APN induces ceramide (Cer) d18:1/24:1 (0.42 ± 0.09 nmol/mg in control-, 0.47 ± 0.10 nmol/mg in HMW-APN- and 0.44 ± 0.10 nmol/mg in LMW-APN-incubated cells), but total Cer levels are not altered (Figure 5C and data not shown). LMW-APN increases Cer d18:1/16:0 (0.28 ± 0.11 nmol/mg in control-, 0.30 ± 0.12 nmol/mg in HMW-APN- and 0.32 ± 0.13 nmol/mg in LMW-APN-incubated cells), Cer d18:1/24:0 (0.53 ± 0.11 nmol/mg in control-, 0.57 ± 0.14 nmol/mg in HMW-APN- and 0.58 ± 0.15 nmol/mg in LMW-APN-incubated cells) and Cer d18:1/24:1 (0.42 ± 0.09 nmol/mg in control-, 0.44 ± 0.10 nmol/mg in HMW-APN- and 0.47 ± 0.10 nmol/mg in LMW-APN-incubated cells), and total Cer (2.25 ± 0.63 nmol/mg in control-, 2.36 ± 0.68 nmol/mg in HMW-APN- and 2.47 ± 0.72 nmol/mg in LMW-APN-incubated cells) is elevated (Figure 5C and data not shown). Hexosyl Cer is not changed by the APN isoforms (data not shown). 

Ceramide is, however, not elevated in the liver of APN knock-out mice on a standard chow, and hexosyl Cer is even reduced [13]. Therefore, *in vitro* findings in PHH are not in accordance with the murine *in vivo* situation. This may be partly related to differences in human and murine cells. APN deficiency in mice does not only affect the liver, but also influences other tissues and cells, and this may be associated with alterations in liver lipids [33]. Nevertheless, current data obtained *in vitro* and experimental evidence from APN-deficient mice and lean mice injected with APN [12,13] indicate that adiponectin receptor-associated ceramidase is specifically activated in obesity.

## 3. Experimental Section

### 3.1. Materials

Dulbecco’s modified eagle medium (DMEM) was from PAA (Karlsruhe, Germany). The RNeasy Mini Kit was from Qiagen (Hilden, Germany), and oligonucleotides were synthesized by Metabion **(**Planegg-Martinsried, Germany). The LightCycler^®^ 480 SYBR Green I Master was purchased from Roche (Mannheim, Germany). Triglyceride concentrations were measured using the Glycerol-3-phosphate oxidase Phenol 4-Aminoantipyrine Peroxidase (GPO-PAP) microtest (purchased from Roche, Mannheim, Germany), and total cholesterol was determined by using an assay from Diaglobal (Berlin, Germany). Flotillin-1 antibody was from BD Transduction Laboratories (Heidelberg, Germany); recombinant APN (HMW-APN) and APN antibody were ordered from R&D Systems (Wiesbaden-Nordenstadt, Germany). LMW-APN was produced in insect cells, as described [16]. The β-actin antibody was from New England Biolabs GmbH (Frankfurt, Germany). Hepatocyte cell lines HepG2, Hep3B and PLC/PRF/5 were obtained from the American Type Culture Collection (Wesel, Germany) and were cultivated in RPMI medium (GIBCO-BRL, Karlsruhe, Germany) supplemented with 10% FCS. Cultivation in the presence of APN was done in serum-free medium.

### 3.2. Primary Human Cells

Non-neoplastic tissue samples from liver resections were obtained from patients (male = 8, female = 7) undergoing surgical liver segment resection or partial hepatectomy for liver tumors or metastatic liver tumors of colorectal cancer (right lobe = 8, left lobe = 1, segment resection = 6). The mean age of the patients was 57.1 ± 18.0 years and the mean BMI was 25.7 ± 4.9 kg/m^2^. All tissue samples were examined by a pathologist, and only histologically non-tumorous tissue was used. Clinical patient documentation included age, sex, medical diagnosis, presurgical medication, liver function tests and alcohol and smoking habits. Patients with hepatitis, cirrhosis or chronic alcohol use were excluded. Experimental procedures were performed according to the guidelines of the charitable state-controlled foundation, Human Tissue and Cell Research (HTCR) [34], with the written informed patient consent approved by the local ethical committee of the University of Regensburg.

PHHs were isolated using a modified two step EGTA/collagenase perfusion procedure, as described in detail previously [35,36]. The viability of isolated PHHs was determined by trypan blue exclusion, and cells with a viability of more than 85% were used for further work.

Per well of collagen-coated 6-well plates, 10^6^ primary human hepatocytes were cultivated in 2 mL DMEM supplemented with 4.5 g/L glucose, 4 ng/mL hydrocortisone, 1.67 mU insulin, 2 mM glutamine, 1% penicillin/streptomycin and 5% fetal calf serum for 48 h. Thereafter, cells were cultivated in DMEM, 1% penicillin/streptomycin. Experiments were terminated by washing the cells with PBS and solubilization of the cells in radioimmunoprecipitation assay lysis buffer (50 mM Tris-HCl (pH 7.5), 150 mM NaCl, 1% vol/vol Nonidet P-40, 0.5% vol/vol deoxycholic acid and 0.1% (vol/vol) sodium dodecyl sulfate).

### 3.3. Quantification of Lipids

Lipids were quantified by direct flow injection electrospray ionization tandem mass spectrometry (ESI-MS/MS) in positive ion mode using the analytical setup and strategy described previously [37]. A precursor ion of *m*/*z* 184 was used for phosphatidylcholine (PC) [37]. A neutral loss of 141 and 277 Da was used for phosphatidylethanolamine (PE) and phosphatidylinositol (PI) [38], respectively. Sphingosine-based ceramides (Cer) were analyzed using a fragment ion of *m*/*z* 264 [39]. Lipid species were annotated according to the recently published proposal for the shorthand notation of lipid structures that are derived from mass spectrometry [40]. Glycerophospholipid annotation is based on the assumption of even-numbered carbon chains only. SM species annotation is based on the assumption that a sphingoid base d18:1 is present. In case the fatty acid composition was not determined, annotation represents the total number of carbons and double bonds. For example, PC 36:4 comprises species like PC 16:0/20:4 or 18:2/18:2.

In total, 13 SM, 3 dihydrosphingomyelin (dihydroSM), 25 PC, 26 PE, 13 PE P-16:0, 13 PE P-18:1, 12 PE P-18:0, 25 PS, 15 LPC, 8 Cer and 2 hexosylceramide (hexosylCer) species were analyzed.

### 3.4. Monitoring of Gene Expression by Real-Time RT-PCR

The mRNA expression was investigated by semiquantitative real-time PCR using SYBR Green. Real-time RT-PCR was performed using the LightCycler^®^ 480 SYBR Green I Master (Roche, Mannheim, Germany), and the specificity of the PCRs was confirmed by sequencing of the amplified DNA fragments (Geneart, Regensburg, Germany). For quantification of the results, the RNA of respective PHH samples was reverse transcribed and cDNA was serially diluted and used to create a standard curve for each of the genes analyzed. The second derivative maximum method was used for quantification with the LightCycler software. Primers to amplify FGF21 were 5’ ACC AGA GCC CCG AAA GTC T 3’and 5’ CTT GAC TCC CAA GAT TTG AAT AAC TC 3’. Primers to amplify activin A, TIMP-1, MMP-9, ABCB4, GLUT2, ApoB and β-actin for normalization were used as described [5,7,8].

### 3.5. SDS-PAGE and Immunoblotting

Proteins (20 µg) were separated by SDS-polyacrylamide gel electrophoresis and transferred to PVDF membranes (Bio-Rad, Munich, Germany). Incubations with antibodies were performed in 5% nonfat dry milk in Tris-buffered saline, 0.1% Tween. Detection of the immune complexes was carried out with the ECL western blot detection system (Amersham Pharmacia, Deisenhofen, Germany).

### 3.6. ELISA

ELISAs to measure activin A, follistatin and apolipoprotein B-100 in supernatants were performed as described [5,7].

### 3.7. Statistical Analysis

Data are presented as the mean ± standard deviation. Statistical differences were analyzed by a paired Student’s *t*-test (MS Excel), and a value of *p* ˂ 0.05 was regarded as significant.

## 4. Conclusions

APN isoform-specific effects have been demonstrated in monocytes, synovial fibroblasts and adipose tissue [16,17,41]. Current data show that HMW-APN and LMW-APN exert common, as well as distinct effects on hepatocytes. Importantly, even opposing activities of these isoforms have been identified. Therefore, beside absolute concentrations of this adipokine, the ratio of HMW-APN to LMW-APN may be important in liver physiology and should be kept in mind when evaluating clinically significant observations in patients.
